# Correction: Flotillins affect LPS-induced TLR4 signaling by modulating the trafficking and abundance of CD14

**DOI:** 10.1007/s00018-024-05288-y

**Published:** 2024-05-30

**Authors:** Orest V. Matveichuk, Anna Ciesielska, Aneta Hromada-Judycka, Natalia Nowak, Ichrak Ben Amor, Gabriela Traczyk, Katarzyna Kwiatkowska

**Affiliations:** 1 Laboratory of Molecular Membrane Biology, Nencki Institute of Experimental Biology PAS, 3 Pasteur St, Warsaw, 02-093 Poland; 2 Laboratory of Imaging Tissue Structure and Function, Nencki Institute of Experimental Biology PAS, 3 Pasteur St, Warsaw, 02-093 Poland

**Cellular and Molecular Life Sciences (2024) 81:191**

In this article the author name Ichrak Ben Amor was incorrectly written as Ichrak Ben Amore.

The original article has been corrected.

